# Is the worst of the pandemic over for Europe?

**DOI:** 10.1016/j.lanepe.2021.100077

**Published:** 2021-03-11

Almost a year ago, on March 11, 2020, WHO declared the spread of COVID-19 a pandemic. At that time, 90% of cases were concentrated in only four countries, three being in Asia—China, South Korea, and Iran. Italy was the first European country to be crippled by the COVID-19 mayhem, which then swept through the entire continent and beyond in successive waves. European countries now rank high in the list of deaths and cases of COVID-19 per capita. In the first wave, western European countries recorded high excess mortality rates, whereas eastern Europe escaped unscathed due to early travel restrictions and lockdowns in the region. This trend shifted in the second wave, and eastern European countries have now recorded even higher excess mortality rates**.** As vaccination campaigns gain momentum across Europe, many people are starting to believe that the worst of the pandemic is now over. However, the emergence of new virus variants, some of which may be more contagious or deadly, is a sobering reminder that the course ahead is still challenging, and that a careful, well balanced, pragmatic implementation plan is needed to leave the worst of the pandemic behind for Europe.

The plan should first aim to expedite mass vaccinations across the continent and to overcome present obstacles of vaccine production and accessibility. The EU bloc of countries currently has only three vaccines at its disposal—from BioNTech/Pfizer, Moderna, and AstraZeneca, as approved by the European Medicine Agency (EMA). The rolling out of these vaccines has been slower than expected due to limited production capacity. While the West has been dismissive of the Russian Sputnik V vaccine, Russia should be applauded for their efforts in making their vaccine available and affordable to countries across the globe. As of February 2021, 21 countries have granted Sputnik V emergency use authorisation, while over a billion doses of the vaccine were ordered for immediate distribution globally. Countries like Hungary, Serbia, Bosnia and Herzegovina, and Belarus, have fared well by the purchase of Sputnik V. Serbia rolled out Sputnik V and China's Sinopharm vaccines alongside the BioNTech/Pfizer jab and now ranks third in vaccination coverage in Europe after Israel and the UK. More vaccines from different providers need to be approved and rolled out to accelerate reaching immunity at the population level. While the bulk of global vaccine doses are manufactured outside of Europe, ramping up their production in the continent will accelerate local distribution. Support by the French pharma company Sanofi to use its German facility to produce BioNTech/Pfizer vaccines for distribution in Europe will partly help to overcome the bottleneck of vaccine production.

In parallel, public health campaigns—commercials, posters, and advertisements—for vaccinations and continued practice of non-pharmaceutical interventions (mask wearing, social distancing, and hand washing) should be adopted by governments as a long-term action plan for COVID-19 mitigation. Such public health campaigns have proven to be a tried-and-tested method, with success in the containment and elimination of polio and the control of measles, mumps, and rubella. Enhanced cross-border and intra-border contact tracing has also been proven effective in controlling the spread of COVID-19 in many countries such as China, Australia, and New Zealand—a strategy that has not been fully exploited by European public health authorities.

Looking further ahead, for all these measures to work effectively, a collective treaty on preparing for present and future pandemics at a regional level, similar to the Influenza Pandemic Preparedness framework comprising both EU and non-EU European countries, would be most impactful in the long term. As of February 2021, the European Commission has presented plans in this direction, with a proposal for a Health Union to bring together the member states to respond collectively to future cross-border health threats. The plan also includes a new EU agency called European Health Emergency Preparedness and Response Authority (HERA), especially responsible for biomedical preparedness. Although a step in the right direction, a plan that is more inclusive of non-EU member countries would have been more beneficial for the whole region.

The rollout of COVID-19 vaccines has put rich countries at a definitive advantage; at the same time, there is the question of ethical and moral responsibilities to support low-income countries with sufficient vaccine jabs. Almost half of the world's vaccine supply to date has been reserved for only 15% of its people. Three-quarters of all vaccinations so far have happened in ten countries that account for 60% of global gross domestic product, whereas 130 countries have yet to administer a single dose. A WHO–EU joint initiative to fund distribution of vaccines in eastern Europe and central Asian countries aims to support the regions in their vaccination efforts but, without vaccines to distribute, the initiative will fail to help the EU's Eastern neighbours. Even the COVAX initiative—co-led by Gavi, CEPI, and WHO, for fair and equitable distribution of vaccines—is barely scratching the surface. With this initiative, 1•3 billion donor-funded doses are being made available to 92 nations eligible for the Gavi COVAX Advanced Market Commitment, targeting only 20% of population coverage by the end of the year. Albeit benevolent and ambitious, this goal will still leave many vulnerable to infection and transmission.

To truly leave the pandemic behind, the world needs the poorest of the poor and the richest of the rich to have equal access to the vaccines, and Europe has a crucial role to play in this philanthropic mission. A reassuring pledge has been made by the world leaders at the virtual G7 meeting on Feb 19, to donate a part of their vaccine supplies to the developing nations. Only time can tell if these promises will be fulfilled.

*The Lancet Regional Health – Europe*

